# *Clematisguniuensis* (Ranunculaceae), a new species from Eastern China

**DOI:** 10.3897/phytokeys.128.33891

**Published:** 2019-07-23

**Authors:** Rong-Bin Wang, Wei-Yong Ni, Wen-Jing Xu, Zheng-Wen Gui, Shou-Biao Zhou

**Affiliations:** 1 College of Life Sciences, Anhui Normal University, Wuhu 241000, Anhui, China; 2 Institute of Chinese Medicine Resources, Anhui College of Traditional Chinese Medicine, Wuhu 241000, Anhui, China; 3 Administration of Guniujiang National Nature Reserve, Huangshan 245617, Anhui, China; 4 School of Resources and Environmental Engineering, Anhui University, Hefei 230601, Anhui, China; 5 Anhui Provincial Engineering Laboratory of Water and Soil Pollution Control and Remediation, Anhui Normal University, Wuhu 241000, Anhui, China

**Keywords:** Anhui, Early diverging eudicots, Ranunculales, Taxonomy

## Abstract

*Clematisguniuensis***sp. nov.**, a new narrowly endemic species of *Clematis*, is described and illustrated from the Huangshan Mountains of Eastern China. A description of *C.guniuensis* is presented along with illustrations, photographs and diagnostic differences between the new species and its putative close allies.

## Introduction

*Clematis* L. (Ranunculaceae) is a large genus of early diverging eudicots, comprising approximately 280–350 species (Tamura 1987, 1995; Johnson 1997; Grey-Wilson 2000; Wang and Li 2005), out of which 147 species are reported in China, 93 of them being endemic to the country (Wang and Bartholomew 2001). The species of *Clematis* are economically important for their chemical properties relating to traditional medicine and also as ornamentals due to their attractive flowers (Grey-Wilson 2000; Wang and Bartholomew 2001). The genus is distributed worldwide, showing a high degree of speciation, with adaptations to a variety of habitats, especially in eastern Asia (Tamura 1993). *Clematis* species also show considerable morphological diversity and plasticity, making the taxonomy and classifications of the genus notoriously difficult (Brandenburg 2000), with different classifications emphasising different morphological characters (e.g. Prantl 1888; Tamura 1995; Johnson 1997; Grey-Wilson 2000; Wang and Li 2005).

During floristic surveys in experimental forestry plots of this Guniujiang National Nature Reserve between 2016 and 2018, a conspicuous species bearing 1-flowered cymes was collected. After thorough comparisons of diagnostic morphological and anatomical features of similar taxa (Lin and Wei 2009; Wang 2004a, 2004b, 2006a, 2006b, 2015a, 2015b; Wang and Bartholomew 2001; Wang and Huang 2014; Wang and Li 2016; Wang and Xie 2007), we have concluded that this specimen belongs to a hitherto undescribed species. We describe this specimen as a new species, presenting a morphological description, illustrations and comments on morphologically related species.

## Material and methods

Measurements and morphological character assessments of the putative new species were undertaken using herborised and living specimens observed in the field or cultivated at the Botanical Garden of Anhui College of Traditional Chinese Medicine. All available specimens of *Clematis*, stored in the following herbaria (acronyms according to Thiers 2017+): IBK, IBSC, N, MO, P, PE, SYS, US and some local herbaria were examined. Images of type specimens were obtained from Tropicos.org (http://www.tropicos.org) and JSTOR Global Plants (http://plants.jstor.org). All morphological characters were studied using a dissecting microscope (SZX16, Olympus, Tokyo, Japan). Characters were described, using the terminology presented by Wang and Bartholomew (2001).

## Taxonomy treatments

### Clematis guniuensis

Taxon classificationPlantaeRanunculalesRanunculaceae

W.Y.Ni, R.B.Wang & S.B.Zhou sp. nov.

4b73558d-4cf7-576a-8f91-8db6b24ccd07

urn:lsid:ipni.org:names:77200428-1

Figs 1 , 2 , 3

#### Diagnosis.

Resembles *C.florida* Thunb. and *C.huchouensis* Tamura but can be distinguished from the former one by puberulous leaflet blades, longer petiole, larger flowers with light green sepals, longer stamens and white filaments and from the latter by its longer petioles, 3-lobed leaflet blades, shorter pedicel, larger flowers, 4 sepals, filaments about 3–5 times the length of the anther, persistent style 1.5–2 cm long, and yellow plumose.

#### Type.

CHINA. Anhui Province: Qimeng County, Guniujiang National Nature Reserve, Huangshan City, 30°0'57.02"N, 117°29'37.17"E, 550 m a.s.l., 15 May 2018, flowering, Rong-Bin Wang, WRB201805068 (holotype: ANUB!; isotypes: AHU!, PE!, WUH!).

#### Description.

Vines herbaceous, perennial; branches inconspicuously longitudinally 6-sulcate to sub-terete, densely primrose yellow puberulous covering when young, becoming glabrescent with age. Root fusiform. Leaves opposite, ternate; petiole 7–10 cm long; leaflets 3-lobed, ovate to narrowly-ovate; central lobe 6–7.5 × 3.5–4 cm, lateral lobes 4–5 × 2.8–3.5 cm, margin coarsely dentate to entire, apex acuminate or sometimes caudate, base broadly cuneate to rounded, papery, adaxially dark green, densely appressed white pilose, abaxially light green, sparsely puberulous to sub-glabrous, basal veins abaxially slightly prominent; and with petiolule 1–2 cm long. Cymes axillary, 1-flowered; peduncles 3–6 cm long, densely puberulous; bracts opposite, subsessile, ovate, 1.2–1.7 × 5–7 mm, margin entire, both surfaces puberulous. Flowers 6–8 cm diam.; pedicels ca. 2 cm long, conical, sulcate, green, densely puberulous; sepals 4, spreading, light green, ovate, ovate-lanceolate or broad-lanceolate, 3.5–4.5 × 1.8–2.3 cm, apex acute, adaxially glabrous, abaxially sparsely white pubescent, trinerved; stamens numerous, 1–3 cm long, filaments linear, glabrous, about 3–5 times the length of the anthers, anthers narrowly oblong, ca. 6 mm long, white, glabrous, apex shortly apiculate; ovaries ellipsoidal, pubescent, style densely yellow-villose. Achenes dark-brown, strongly compressed, ovate to broadly ellipsoidal, ca. 3 × 1 mm wide, pubescent; persistent style 1.5–2 cm long, yellow-plumose.

**Figure 1.**
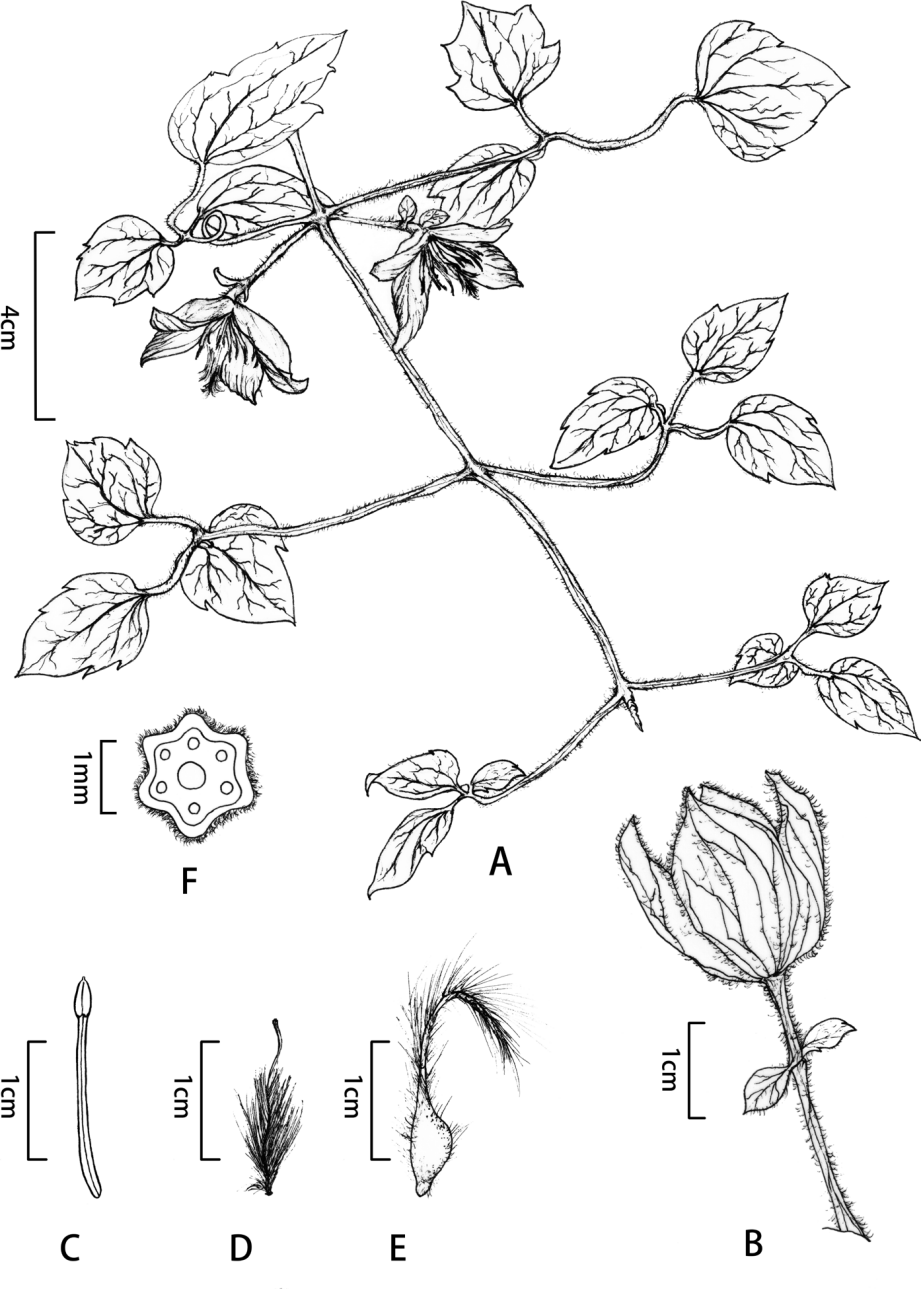
*Clematisguniuensis* W.Y.Ni, R.BWang & S.B.Zhou. **A** Habitat in flowering period **B** Inflorescences with budding flower, showing the bracts **C** Stamen **D** Pistil **E** Achene **F** Stem cross-section.

**Figure 2.**
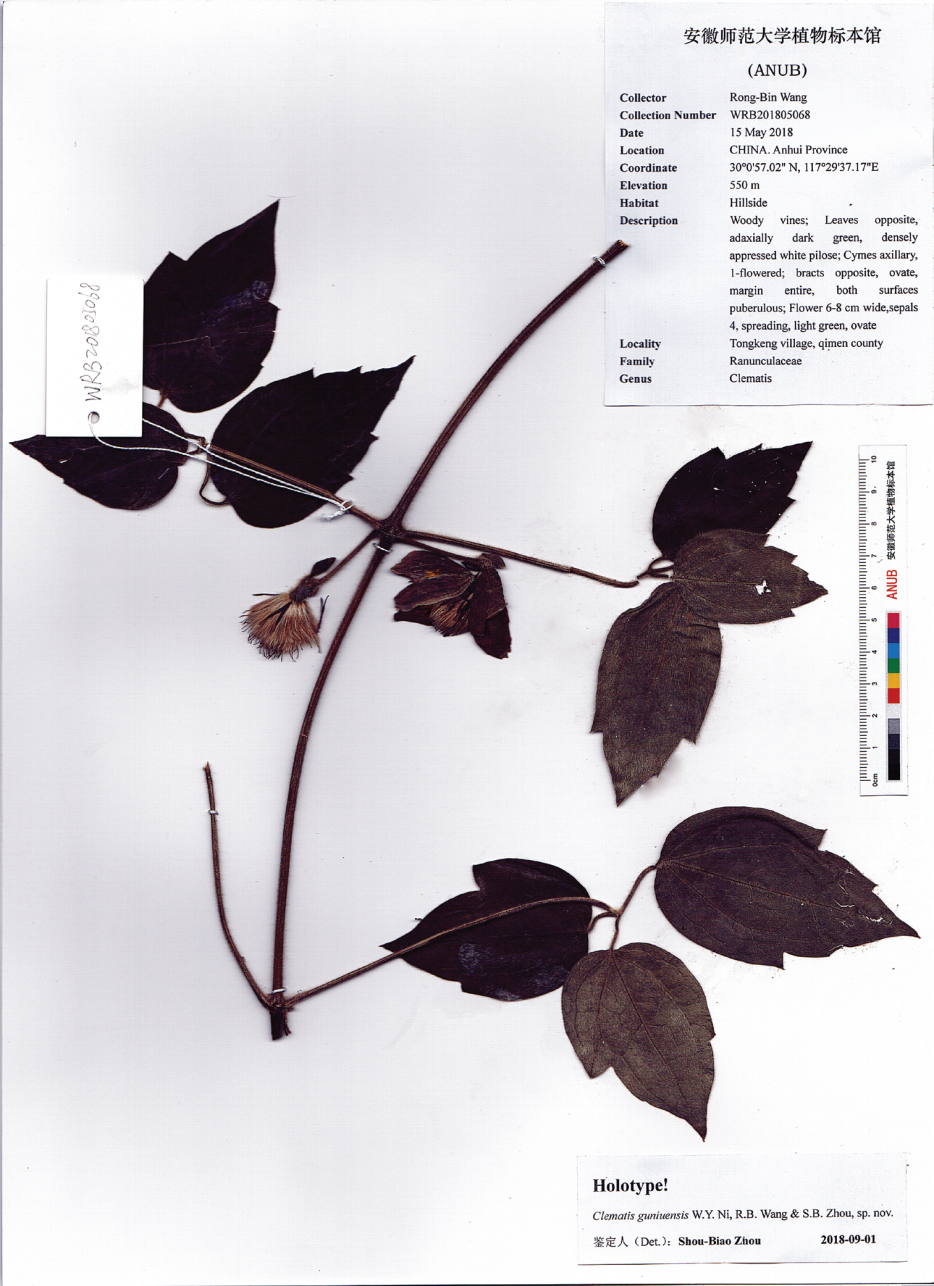
Holotype of *Clematisguniuensis* W.Y.Ni, R.B.Wang & S.B.Zhou.

**Figure 3.**
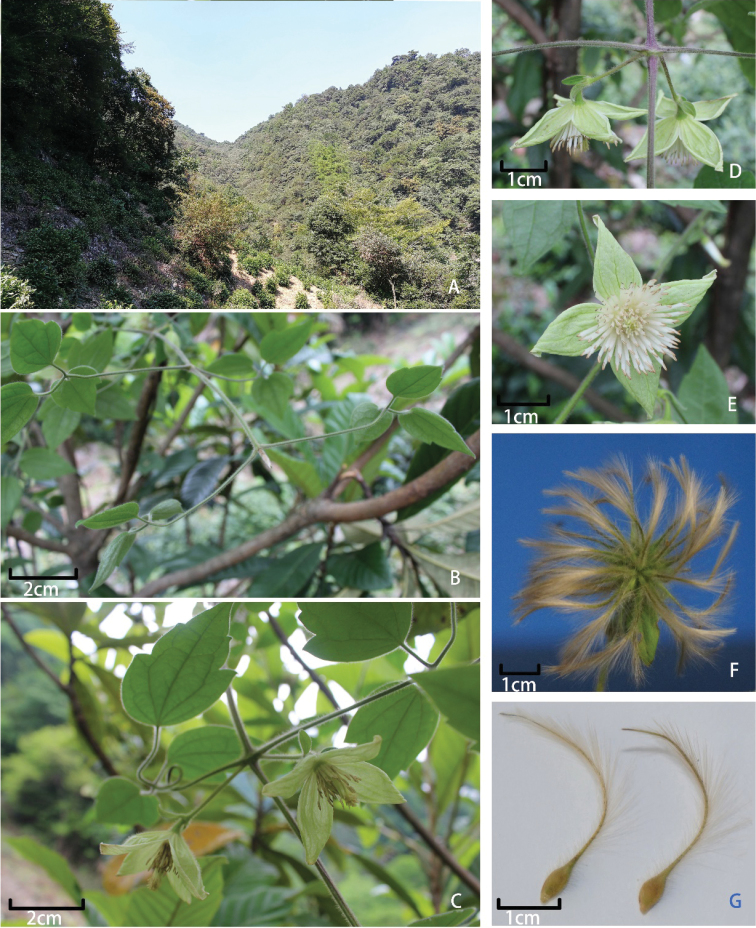
*Clematisguniuensis* W.Y.Ni, R.B.Wang & S.B.Zhou. **A** Habitat **B** Young branches, showing stems 6–grooved, puberulous **C** Inflorescences, showing style and abaxial surface view of leaf blade **D** Dorsal view of cymes, showing peduncles and bracts **E** Frontal view of flower, showing stamens **F** Fruit **G** Achenes, showing persistent style.

#### Phenology.

Flowering from April to May; fruiting from October to November.

#### Etymology.

The specific epithet is derived from the type locality, Guniujiang National Nature Reserve.

#### Vernacular name.

Gǖ Niǘ Tiě Xiãn Lián (Chinese pronunciation); 牯牛铁线莲 (Chinese name).

#### Distribution and habitat.

**Figure 4.**
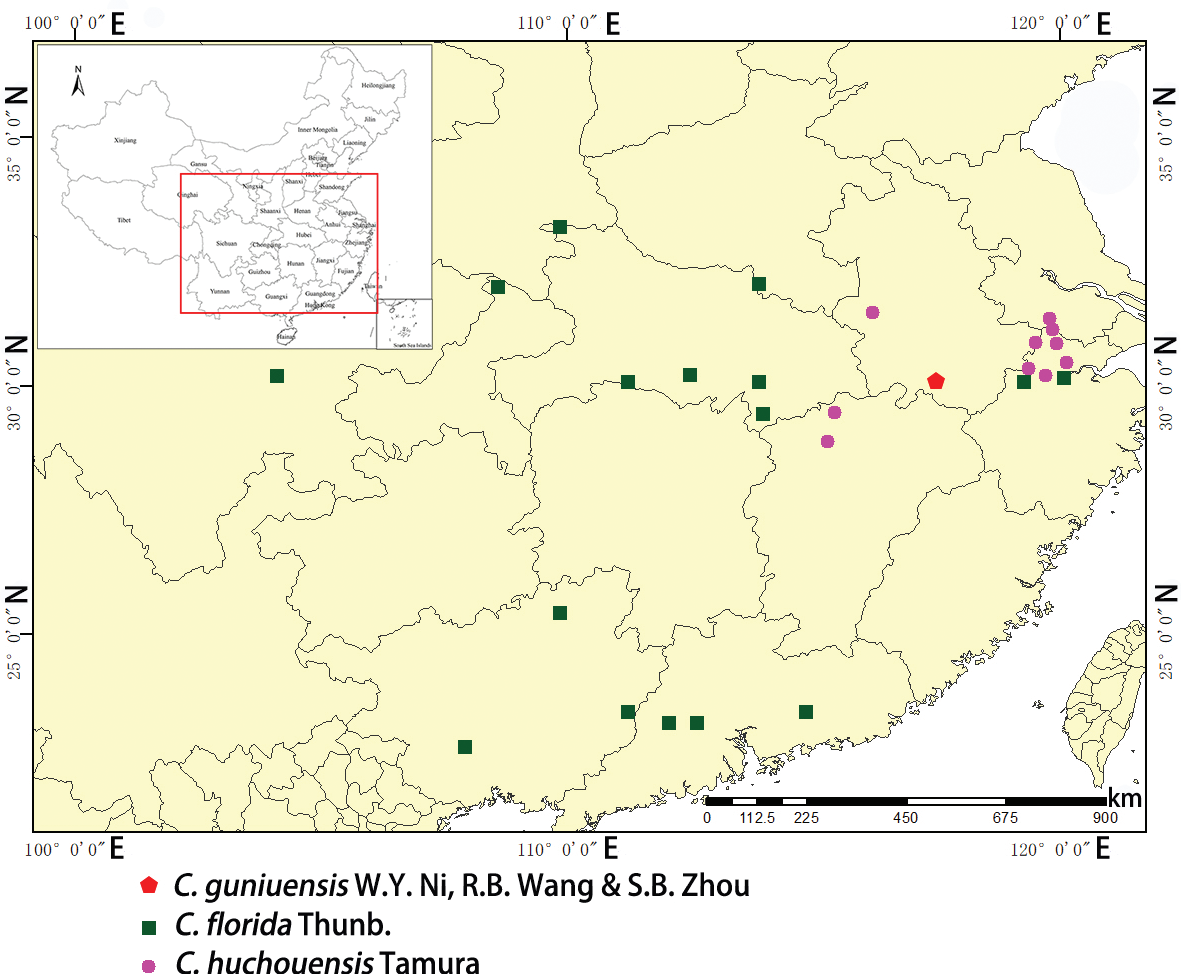
Distribution map of *Clematisguniuensis* (pentagon, red) and its congeners *C.huchouensis* (round, violet) and *C.florida* (square, green).

To date, *C.guniuensis* is only known from the type locality, Guniujiang National Nature Reserve, Huangshan City, Anhui Province (Fig. 4). Currently the species is known from a few collections and there is only one known population with ca. 20 individuals at the type locality. The species is mostly found in tea plantations or forest edges along valleys of evergreen broad-leaved forests, at an elevation of 1,500 m a.s.l.

#### Conservation assessment.

Based on the present field investigations, *C.guniuensis* is currently only known from the type locality and with a very small population size (ca. 20 individuals). The species should be given the IUCN status of Critically Endangered (CR) based on criteria D: “Population size estimated to number fewer than 50 mature individuals” (IUCN 2016).

#### Notes.

A morphological comparison between *C.guniuensis* and morphologically related species, *C.florida* and *C.huchouensis*, is provided in Table 1. A total of 17 species of this genus was found in the Anhui province, with this new species being easily distinguished from the other species in this region by its 3-lobed leaflets, 1-flowered cymes, flowers 6–8 cm diam., sepals 4 and light green and glabrous filaments.

**Table 1.** Diagnostic character differences amongst *Clematisguniuensis*, *C.huchouensis* and *C.florida*.

Characters	* Clematis guniuensis *	* Clematis huchouensis *	* Clematis florida *
Petioles	7–10 cm long	1.7–3 cm long	2–4 cm long
Shape of leaflet blades	3-lobed	2- or 3-lobed or undivided	undivided
Indumentum of leaflet blades	puberulous	puberulous	glabrous
Flower per cyme	1-flowered	1–3-flowered	1-flowered
Size of bracts	1.2–1.7 cm long	2–3 cm long	1.4–3 cm long
Size of pedicels	ca. 2 cm long	1.2–3 cm long	3.7–8.5 cm long
Size of flowers	6–8 cm wide	2–3 cm wide	3.6–5 cm wide
Number of sepals	4	4	6
Colour of sepals	light green	white	white
Size of sepals	3.5–4.5 ×1.8–2.3 cm	1.4–2.2 × 0.3–0.6 cm	2–3 × 1–1.5 cm
Size of anthers	ca. 6 mm long	2.5–3.2 mm long	2.5–3.5 mm long
Filaments	about 3-5 times the length of the anther, white	equal to the length of the anther, white	shorter than anthers, purple
Persistent styles	1.5–2 cm long, yellow plumose	0.8–1.3 cm long, appressed yellowish pubescent	ca. 8 mm long, basally spreading puberulous, apically glabrous

## Supplementary Material

XML Treatment for Clematis guniuensis

## Appendix 1

List of all specimens analyzed for all three species for this study, with voucher number and deposition and origin information. Species are listed in alphabetical order.

***Clematisflorida*** Thunb.

**CHINA. Anhui Province**: Zhu P.Z., *64-0067* (JSPC); Ho Y.Y., *Y.Y.Ho-02243* (SYS); **Chongqing City**: Liu Z.Y., *Liu Z.Y.-972413* (IMC); **Guangdong Province**: Huang C., Huang-162703 (IBSC); Chen W.Y., *Chun Woon Young 8416* (PE); Chun Y.F., *Y.F.Chun-30488* (SYS); Shi G.L., *Shi G.L.13249* (WUK); **Guangxi Zhuang Autonomous Region**: Mashan Expedition Team, *450124140722047LY* (GXMG); Liu Y. et al., *Liu & Yu H0094* (IBK); Guangxi Investigation Team, *Guangxi-3984* (PE); **Guizhou Province**: Huang W.L. & Tu Y.L., *Huang & Tu-0459* (GNUG); Wang Z.R., *522628140720152LY* (GZTM); Gao J., *Gao2013233024* (QNUN); Li K., *DPS2013233024* (QNUN); **Henan Province**: Ye Y.X., *20071024B8* (BJFC); Li G.Q., *Li4257* (HEAC); Yang W.G., *Yang W.G.58* (HEAC); Xu C.X., *Xu 78055* (HENU); Yang X.F., *Yang 28* (HENU); Wang X.F., *Wang 181* (HENU); Fu J.Q., *Fu 2201* (IBK); **Hubei Province**: Wu J.Q. & Ma X.Y., *Wu & Ma 8061* (HIB); **Sichuan Province**: Li J.Y., *ss201208040024* (BJFC); Chien Y., *Y.Chien 5993* (N); Yan J.X., *Yan 87029* (PEM); Chien S.S., *Chien Sung Shu 5342* (SZ); **Zhejiang Province**: Cheng R.C., *R.C. Cheng 1976* (IBSC); Rwan Chia Tsuin, *Rwan Chia Tsuin 37* (N); Zhejiang Investigation Team, *Shen Jia-Yu 8065* (NAS).

***Clematishuchouensis*** Tamura

**CHINA. Guizhou Province**: Huo P.Z., Zhi M.G. & Yuan D.X., *520222150126001LY* (GZTM); **Jiangsu Province**: Wu W.X., *Wu W.X.4205* (NAS); Ding Z.Z. & Wang Y.C., *Ding & Wang* 0939 (NAS); Wu W.X., *W.X.Wu 6087* (NAS); Wu W.X., *Wu W.X.4351* (WUK); **Jiangxi Province**: Shen S.J., *Shen S.J.381* (PE); Shen S.J., *Shen S.J.*00565 (PE); **Sichuan Province**: Ju W.B. & Deng H.N., *HGX13374* (CDBI); **Zhejiang Province**: Jiang Y.P., *HZ008256* (HHBG); Tu Z.B., *Tu0104710* (HIB); Chen S., *S.Chen 1931* (NAS); Chen M., *M.Chen 868* (NAS); Zhang S.R., *Zhang S.R.1094* (PE).
